# Numerical Study on Electromagnetic Hydraulic Forming Process to Overcome Limitations of Electromagnetic Forming Process

**DOI:** 10.3390/ma17071586

**Published:** 2024-03-30

**Authors:** Yeon-Bok Kim, Jeong Kim

**Affiliations:** Department of Aerospace Engineering, Pusan National University, Geumjeong-gu, Busan 46241, Republic of Korea; 941208b@pusan.ac.kr

**Keywords:** Electromagnetic Forming (EMF), Electromagnetic Hydraulic Forming (EMHF), finite element analysis (FEA), high-speed forming

## Abstract

This paper provides a comparison between the conventional Electromagnetic Forming (EMF) technique and the novel Electromagnetic Hydraulic Forming (EMHF) approach. The EMHF involves the use of finite element analysis coupled with the EM and arbitrary Lagrangian–Eulerian techniques analyzed through LS-DYNA. In the free-bulge configuration, EMF is influenced by the forming coil, resulting in a dead zone and uneven forming. Additionally, EMF can only be used to shape materials with high electrical conductivity. In contrast, EMHF, driven by induced hydraulic pressure from the electromagnetic field-affected drive sheet, is independent of the electrical conductivity of the material and produces dome-shaped workpieces. For rectangular die shapes, EMF is prone to collision owing to the acceleration of the blank, which results in a reduced quality owing to bouncing. However, EMHF exhibits no bouncing effect and successfully achieves the target shape in most cases. The two techniques differ in the strain rate, with EMF at 4850/s, whereas EMHF operates at approximately 1250/s. Despite being slower, EMHF is still a high-speed forming technique. In conclusion, EMHF is a promising technique capable of addressing the shortcomings of conventional EMF and achieving improvements in forming processes.

## 1. Introduction

High-speed forming enables a blank to be shaped within a short duration of 1 ms or less, resulting in enhanced formability. This allows for the formation of complex shapes compared with low-speed forming methods. Moreover, it provides the advantage of reducing spring back due to inertial effects [1,2,3]. High-speed forming techniques include explosive forming, EMF (Electromagnetic Forming), and EHF (Electrohydraulic Forming). Explosive forming is a technique that utilizes high-impact pressure generated from an explosion to shape metal plates [4,5,6]. Explosives release their energy instantaneously, resulting in extremely high pressure for a short duration at the moment of detonation. However, the use of explosives in experiments raises safety concerns. EMF employs powerful electromagnetic forces to induce eddy currents directly into the workpiece, allowing shaping of the product without the need for mechanical contact, which is one of its notable advantages [7,8,9]. However, the electrical conductivity of the material significantly affects the process, making it impossible to select materials such as steel or titanium. EMF depends on the forming coil. Forming coils are typically used for compression, tension, and sheet metal forming [10,11]. Among them, the spiral type is primarily used in sheet metal forming because it vertically distributes the Lorentz force. However, a dead zone exists at the center of the forming coil, resulting in an uneven distribution of the Lorentz force on the blank. Furthermore, because the blank is processed by the Lorentz force generated within the blank itself rather than by external objects, the forming speed of the workpiece can result in a bouncing effect due to collisions with the die of the target shape, leading to unsatisfactory forming outcomes. Efforts are being made to address the inherent issues in forming by developing coils of the uniform pressure actuator (UPA) type [12,13,14], aimed at achieving a uniform distribution of the Lorentz force. However, resolving fundamental problems, such as the bouncing effect, remains challenging. EMF is increasingly utilized in shearing, reduction, and tube expansion processes rather than forming, primarily because of these limitations [15,16,17]. EHF generates intense plasma within a chamber to form material using instantaneous hydro-pressure from an explosion [18,19,20]. This is not influenced by the electrical conductivity of the material compared with that of EMF, and there is no dead zone, making it superior in terms of the forming results. However, the forming process is significantly influenced by the location of the plasma generated by the load. Because the plasma position is fixed, producing asymmetric workpieces is challenging. In addition, in practical applications, the time-consuming process of replacing and securing consumable wires poses a significant challenge. This is a productivity drawback that requires further improvement. In addition, there are several other high-speed forming techniques, each with its own advantages and disadvantages. However, further studies are required to improve these methods.

Electromagnetic Hydraulic Forming (EMHF) combines the strengths of EMF and EHF techniques. EMF offers the advantage of instantaneously generating powerful forces leading to superior productivity and speed. Conversely, EHF utilizes fluid as a load, eliminating the need for custom forming coils according to the target shape and allowing for a more uniform load distribution. In EMHF, an electromagnetic field is applied to a drive sheet made of a highly conductive material, which drives the punch. This process achieves instantaneous forming by swiftly pushing the water in contact with the punch at high speeds.

In this study, a finite element analysis using LS-DYNA was conducted to evaluate the formability of materials with EMHF technology. To evaluate the technical feasibility of EMHF, we performed a comparison between the EMF and EMHF techniques using Al5052 and SUS430 materials in a free-bulging die setup. Furthermore, the differences between the two techniques were compared based on the analysis results for specific die shapes. Through an assessment of EMHF formability, we confirmed its potential as a viable alternative to other high-speed forming methods.

## 2. Numerical Modeling in LS-DYNA

### 2.1. Electromagnetic Forming Modeling

**Figure 1:**
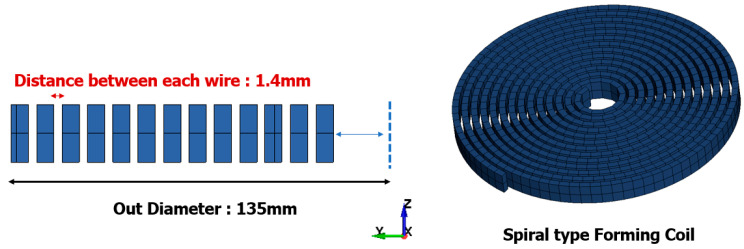
Specifications and shape of forming coil.

LS-DYNA [21] is a finite element program capable of structural analysis using electromagnetic fields. This is facilitated through the “EM” keyword [22,23,24], which operates based on Maxwell’s equations [25]. The analysis model consists of the necessary components for the forming process, including the forming coil, blank, die representing the target shape, and holder for the blank. The forming coil used in this study was spiral-type, with a diameter of 13.5 mm and 13 turns. Its dimensions were 3 × 10 mm, as depicted in Figure 1.

**Figure 2:**
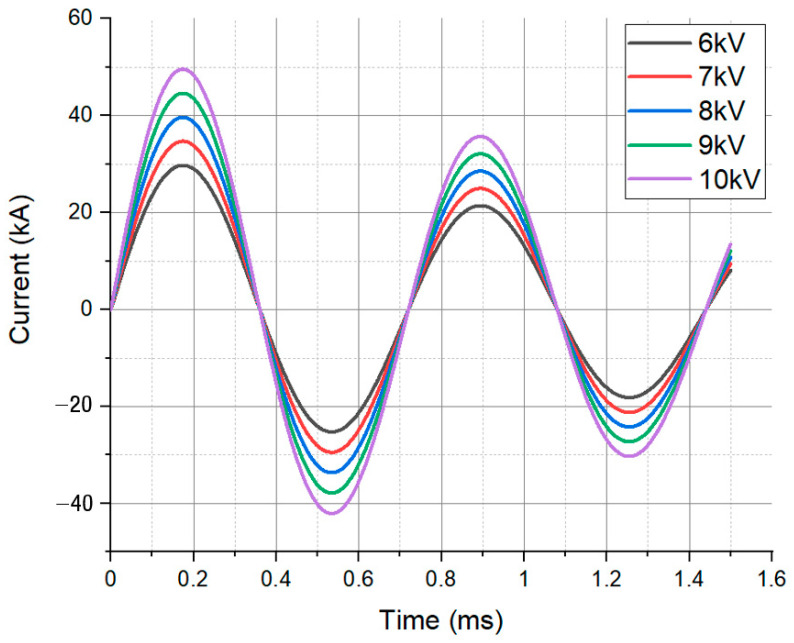
Current flowing through the forming coil at different voltages.

Figure 2 shows the current graph obtained from the coil in the EMF system. The EMF system contains an RLC circuit, and Equations (1)–(4) enable us to derive the current graph [26,27,28].

(1) $$\frac{d^{2}I\left(t\right)}{dt^{2}}+2\xi \omega \frac{dI\left(t\right)}{dt}+\omega^{2}I\left(t\right)=0$$

(2) $$I\left(t\right)=\frac{V_{0}\sqrt{C/L}}{\sqrt{1-\xi }}e^{-\xi \omega t}\sin\left(\omega t\right)$$

(3) $$\xi =\frac{1}{2}R\sqrt{C/L}$$

(4) $$\omega =\sqrt{1/LC}$$

**Figure 3:**
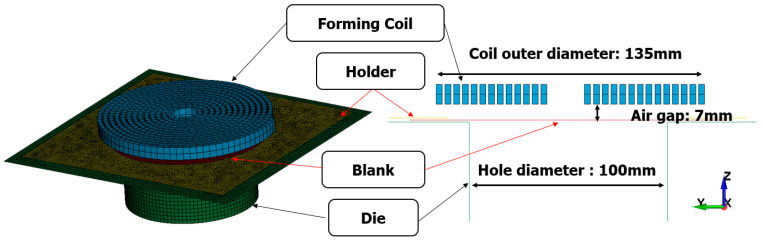
Schematic of EMF model in LS-DYNA.

In the context provided, I represents current, $V_{0}$ denotes the initial input voltage, ω stands for frequency, ξ represents the damping coefficient, t indicates time, R denotes resistance, L signifies inductance, and C represents capacitance. In this study, the values used were R = 0.02 Ω, L = 21.9 μF, and C = 333 μF. In EM analysis, important keywords are RLC curves and electrical conductivity of the material. The coil, serving as the Lorentz force, is primarily made of copper, which has excellent electrical conductivity, with a value of $5.85\times 10^{7}/\Omega m$ being utilized. The blank experiences eddy currents due to the influence of the coil, and the electrical conductivity values are input based on the materials used. In this study, the materials utilized were Al5052 and SUS430, with respective conductivity values of $2.004\times 10^{7}/\Omega m$ and $0.2\times 10^{7}/\Omega m$. The remaining holder and die have minimal impact on the actual forming process, thus insulator properties were employed. The air gap between the forming coil and the blank significantly affects the generation of Lorentz force. In this case, there was a 7 mm gap between the blank and the coil (Figure 3). The blank was used with dimensions of 180 × 180 mm and a thickness of 1 t.

In the EMF analysis, a solid-type coil was used, while the blank, holder, and die were of a shell type. Since the coil, holder, and die didn’t have a significant impact on the actual experiment, the keyword ‘MAT_020-RIGID’ was utilized. For the blank, the keyword ‘MAT_024-PIECEWISE_LINEAR_PLASTICITY’ was used. The analysis on the plastic region can be implemented by inputting curves considering strain rate.

(5) $$\sigma =\left(A+B\varepsilon^{n}\right)(1+{\left(\frac{\dot{\varepsilon }}{C}\right)}^{\frac{1}{p}})$$

(6) $$\sigma =\left(K\varepsilon^{n}\right)(1+{\left(\frac{\dot{\varepsilon }}{C}\right)}^{\frac{1}{p}})$$

**Figure 4:**
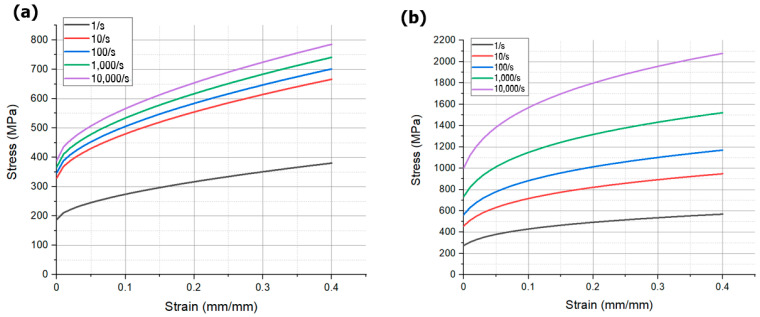
True stress and strain curves at different strain rates: (**a**) Al5052, (**b**) SUS430.

In this equation, σ$\left(\mathrm{MPa}\right)$ is the equivalent stress, ε $\left(\mathrm{mm}/\mathrm{mm}\right)$ is the equivalent plastic strain, and ε$\left(/s\right)$ is the dimensionless strain rate. The material constants are A, B, K, n, C, and p. A is the yield stress of the material, B is the strain hardening constant, n is the strain hardening coefficient, and C is the strengthening coefficient of strain rate. Both equations are based on the Cowper–Symonds model, with different semi-empirical data. Equation (5) was employed for Al5052, while Equation (6) was utilized for SUS430. Specific values are presented in Table 1 and Table 2, and Figure 4 depicts the curves corresponding to strain rate.

### 2.2. Electromagnetic Hydraulic Forming Modeling

**Figure 5:**
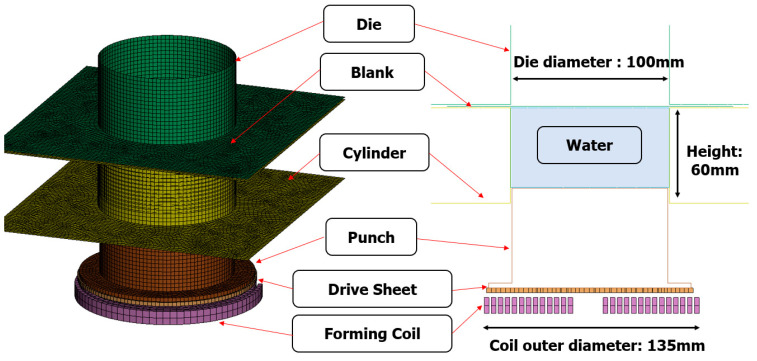
Schematic of EMHF model in LS-DYNA.

EMHF analysis in LS-DYNA employs the “EM” keyword for implementing electromagnetic field analysis and the “ALE” keyword for implementing fluid–structure analysis [29,30]. However, it is challenging to integrate EM and ALE analysis techniques into a single step. Therefore, a two-step analysis was conducted using EM analysis to accelerate the punch through the electromagnetic force generated in the coil, followed by the application of the ALE technique to shape the material using hydraulic pressure. To compare the formability of the EMF and EMHF techniques, analyses were conducted using the same forming coil and applied voltages. The EMHF model consisted of a forming coil, drive sheet, punch, cylinder, blank, die, and water. The models are shown in Figure 5. The drive sheet was aluminum, which is known for its excellent electrical conductivity, and employed the same free bulge die used in a previous EMF analysis. The coil and blank were both utilized with identical dimensions and input values for EMF analysis.

In LS-DYNA, there are various methods for implementing water or fluid using the arbitrary Lagrangian–Eulerian (ALE) technique. In this study, water was implemented using the VOLUME_FRACTION_GEOMETRY keyword, and the equation of state (EOS) was input using the LINEAR_POLYNOMIAL keyword.

**Figure 6:**
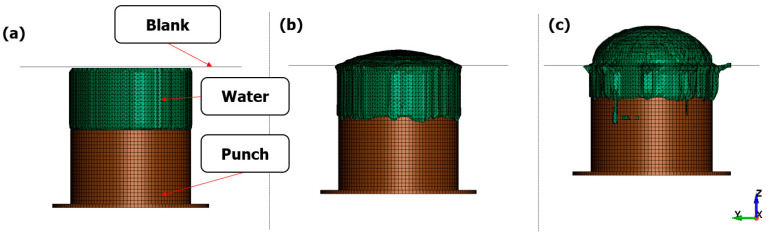
Water flow during EMHF: (**a**) 0 ms, (**b**) 0.7 ms, and (**c**) 1.4 ms.

Figure 6 illustrates the process of forming the punch as it pushes water while being displaced due to electromagnetic forces, as analyzed through electromagnetic analysis. In the initial analysis stages, water was implemented using the ALE keyword, and structural analysis of the punch, water, and blank was performed. Additionally, no external leakage occurred from the blank during the forming of water, indicating that coupled analysis was performed successfully. We restricted the input voltage graph to 8 kV to compare the forming shapes under the same input voltage conditions for the Lorentz force.

## 3. Comparison with EMF and EMHF in Finite Element Analysis (FEA)

### 3.1. Free-Bulge Analysis

**Figure 7:**
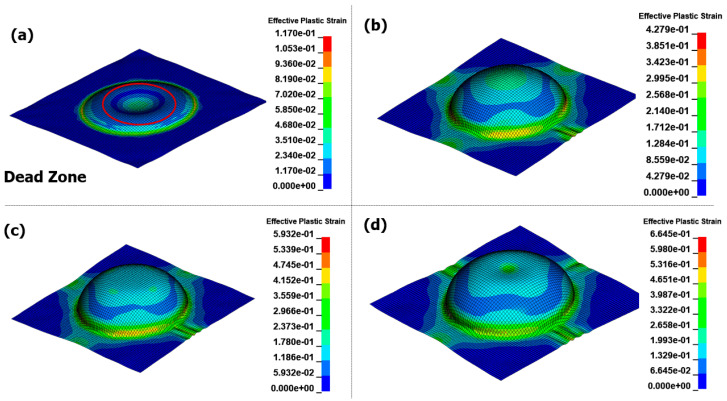
Effective plastic strain for EMF in different times with 8 kV input voltage: (**a**) 0.12 ms, (**b**) 0.25 ms, (**c**) 0.5 ms, and (**d**) 1.0 ms.

**Figure 8:**
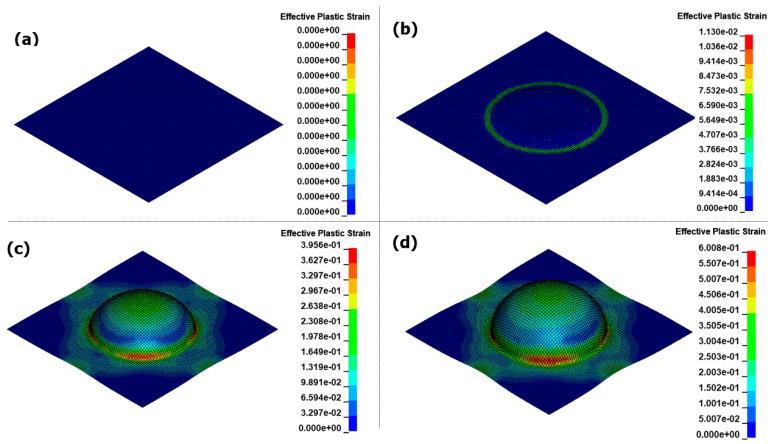
Effective plastic strain for EMHF in different times with 8 kV input voltage: (**a**) 0.12 ms, (**b**) 0.35 ms, (**c**) 1.0 ms, and (**d**) 1.6 ms.

There was a significant difference in the forming path of the blank in the two methods. In the EMF method, forming is achieved by the Lorentz force acting on the blank, where eddy currents flow. Figure 7 illustrates the shape of the blank at various times during the analysis. As depicted in Figure 7a, because the current does not flow at the center of the forming coil, the Lorentz force does not act on the center of the blank, creating a dead zone. The area with the highest forming force is typically found at intermediate positions away from the center of the blank, where the majority of the forming process is facilitated. At this moment, the central region of the workpiece experiences uneven deformation because it is being drawn by the intermediate sections, as shown in Figure 7b. Figure 7c,d represents the repeated results of the shaping analysis after convergence has been achieved. The advantage of the EMF process lies in its ability to perform non-contact shaping on materials with excellent electrical conductivity. However, a drawback is the lack of shaping force due to the minimal occurrence of eddy currents in the center of the material. In other words, there is a disadvantage in that the material vibrates due to the inertia caused by its momentary high-speed motion, and there is no medium to mitigate this. By contrast, the EMHF involves an accelerated punch driven by the Lorentz force while shaping the material by displacing water. The punch is initially rapidly accelerated owing to the influence of the forming coil. However, as it interacts with the fluid and undergoes deformation, its velocity decreases, owing to the reduction in momentum and the conversion of energy into deformation energy (Figure 8). In the interpretation results, it can be observed that while the EMF process is nearing completion and converging, the EMHF process begins shaping (Figure 8b). Even with the strong Lorentz force generated from the same coil, there is a slight delay in transmitting the shaping force to the material because the fluid and punch need to be pushed. When comparing the results of the Al5052 workpiece, while the EMF exhibited uneven shapes, the EMHF generally achieved a shape closer to that of a dome. In the EMHF results, a significantly high value of effective plastic strain was obtained, indicating a high likelihood of fracture occurring during the experiment. However, this problem can be addressed by adjusting the applied voltage to reduce the forming force.

**Figure 9:**
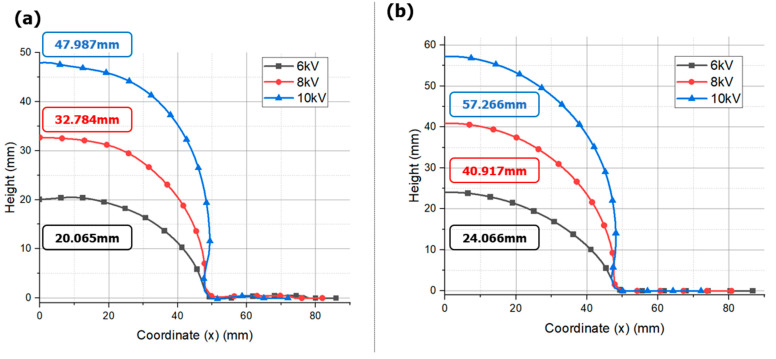
Comparison displacement results with different voltage conditions: (**a**) EMF, and (**b**) EMHF.

Figure 9 compares the displacement values from each analysis model when the input voltage is set to 6 kV, 8 kV, and 10 kV. As the input voltage increases, the bulge height also increases, and it can be observed that the shape of EMHF is more uniform than EMF. This is because the forming force in EMF is not evenly distributed throughout the blank, resulting in uneven forming at the center of the blank. However, EMHF is considered to have superior forming due to the uniform pressure of water acting on the material.

**Figure 10:**
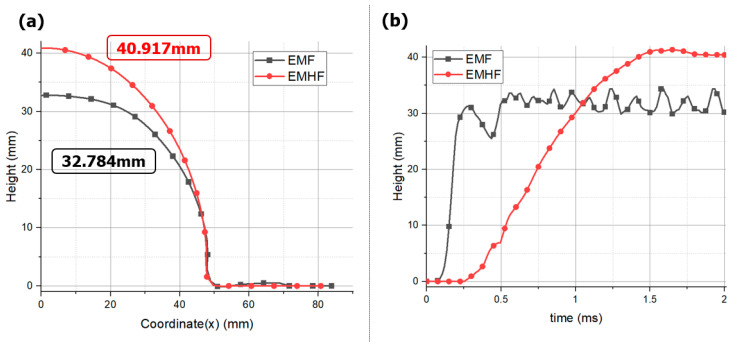
Comparison between EMF and EMHF of (**a**) displacements, (**b**) time graph with 8 kV input voltage condition.

Notably, under the same applied voltage, the state of the formed product and the maximum achievable forming height were superior in EMHF compared to EMF (Figure 10a). The EMF resulted in a more wrinkled shape, whereas the EMHF yielded a shape closer to a dome. This is consistent with the findings from a study by Niaraki, where experimental evidence demonstrated that under identical applied voltage conditions, the EMHF method outperformed EMF [31]. EMHF was measured at 40.917 mm, while EMF was measured at 32.784 mm, indicating that EMHF had an overall higher influx compared to EMF. EMF exhibits a phenomenon where, due to the rapid motion of eddy currents at points, it forms folds at the die inlet. This is essentially a shaping result that can block the influx of specimens [see reference]. In contrast, EMHF undergoes shaping from the center of the specimen upwards, making influx easier. Therefore, it is inferred that EMHF would yield higher shaping results compared to EMF. In our analysis, the forming processes of the two methods converged at different time intervals (Figure 10b). In the case of EMF, forming converges at approximately 0.5 ms, indicating a dominant influence of the initial cycle of the RLC circuit, as demonstrated in Figure 2. On the other hand, the forming convergence of the EMHF occurred at approximately 1.4 ms, indicating a later onset of forming.

**Figure 11:**
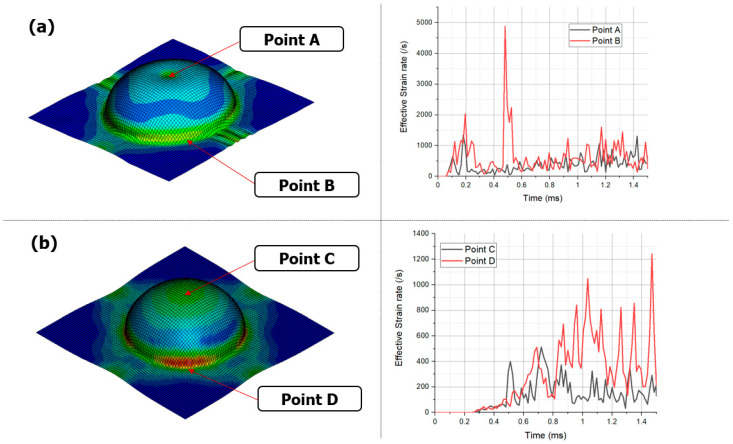
Plastic strain rate at points with time: (**a**) EMF and (**b**) EMHF with 8 kV input voltage condition.

When comparing the strain-rate profiles in the blank during the forming process, a distinct difference was observed. In the EMF analysis, the strain rate values were approximately 1400/s at point A and approximately 4850/s at point B (Figure 11). In contrast, in the EMHF analysis, strain rates of approximately 550/s at point C and approximately 1250/s at point D were measured, which are positions similar to points A and B, respectively. In the EMF, the electromagnetic field generated in the forming coil directly induces eddy currents in the workpiece, resulting in a relatively strong force acting on it. However, in the EMHF, the electromagnetic force from the forming coil leads to momentum conversion in the punch and water, as well as energy conversion during the forming of the workpiece. Consequently, the forming speed of the workpiece tended to be slower. Nevertheless, despite this, obtaining a maximum strain rate of 1250/s indicates that the process falls within the category of high-speed forming techniques.

**Figure 12:**
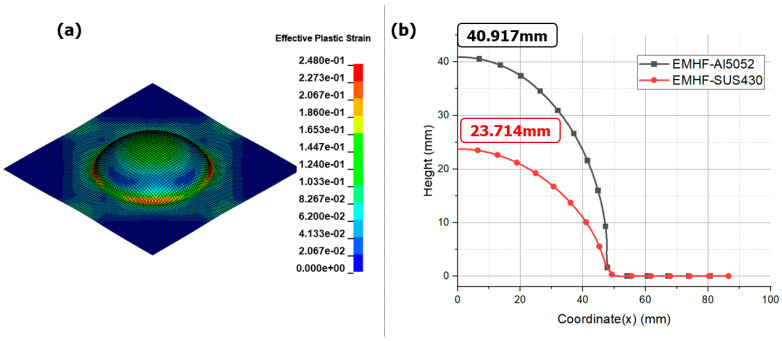
Effective plastic strain for EMHF in different materials with 8 kV input voltage: (**a**) SUS430 blank, (**b**) graph in Al5052 and SUS430.

Finally, the significant difference was more pronounced in terms of the material properties. Figure 12 compares the EMHF forming results for SUS430 with those for Al5052. The EMF technique showed almost no forming, whereas it was evident that forming was feasible using EMHF. The electrical conductivity of SUS430 (1.0 × 10^6^ S/m) is significantly lower than that of Al5052 (2.004 × 10^6^ S/m). In the EMF, the blank follows the governing Maxwell’s equations, where the electrical conductivity of the blank and Lorentz force exhibit an essential correlation. Therefore, for materials with low electrical conductivity, such as steel and titanium, minimal forming is observed. In EMF-related studies, to shape materials with low electrical conductivities, a drive sheet is used to perform simultaneous forming. The drive sheet, primarily composed of highly conductive aluminum, applies a forming force to the target blank [32,33]. However, a drawback is that a drive sheet is required for each forming operation, and it cannot be reused because it is considered a consumable. In contrast, the EMHF operates independently of the electrical conductivity of the workpiece, with the fluid pressure being the predominant factor. This suggests that the forming technique can be applied to a wide range of materials to overcome the limitations of EMF.

### 3.2. Rectangular Die Analysis

**Figure 13:**
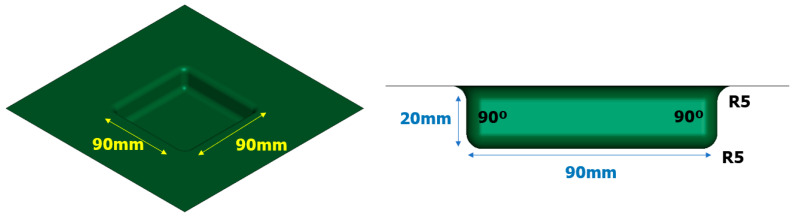
Shape of the rectangular die for EMF and EMHF analysis.

In order to assess the practical applicability of the EMHF technique, an analysis was conducted on a die with a specific shape. Figure 13 illustrates a simplified model of a square-shaped die with a diameter of 90 × 90 mm and a 5 mm radius, represented as a shell shape. An applied voltage of 8 kV was used following the current graph used in the previous free-bulge analysis.

**Figure 14:**
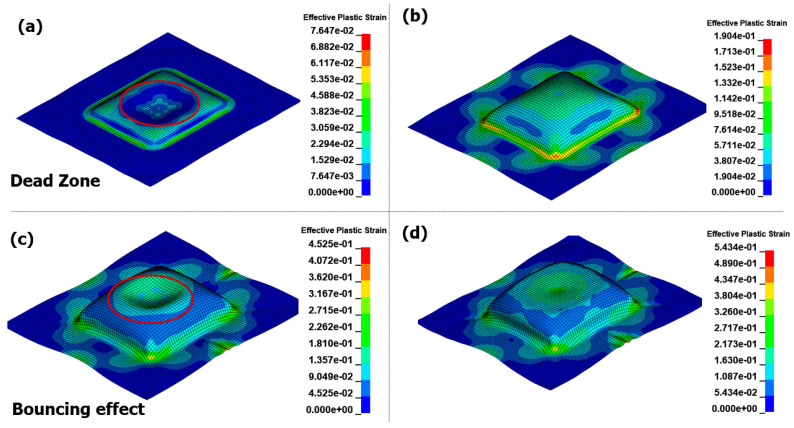
Effective plastic strain for EMF in different times with 8 kV input voltage: (**a**) 0.1 ms, (**b**) 0.18 ms, (**c**) 0.35 ms, and (**d**) 1.0 ms.

Figure 14 illustrates the results of the EMF analysis over time. During the initial forming stage of the EMF process, eddy currents flow through the material, and the Lorentz force acts upon it. The region in the center, where no current can be generated from the forming coil, is referred to as the dead zone. At 0.1 ms, the surrounding intermediate section experiences a strong electromagnetic force, likely resulting in uneven flow, as it predominantly pulls most of the forming. At 0.18 ms, contact with the die occurs at a height of 20 mm for the first time. As the EMF causes forming through the motion of the blank without external action, collisions with the die occur at high speeds. This leads to a significant reaction force between the die and the blank, known as the bouncing effect. After forming convergence at 0.35 ms, even if the current flows through the coil, the significant difference in the air gap between the blank and coil prevents the implementation of the Lorentz force required for forming. In the final result, the bouncing effect causes surface irregularities and prevents forming flow at the die edge, posing significant challenges for the application of EMF in sheet metal forming.

**Figure 15:**
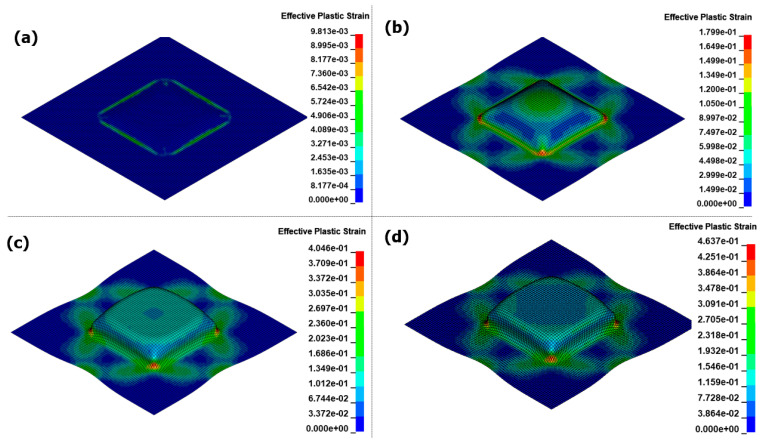
Effective plastic strain for EMHF in different times with 8 kV input voltage: (**a**) 0.4 ms, (**b**) 0.78 ms, (**c**) 1 ms, and (**d**) 1.35 ms.

Figure 15 shows the results of the EMHF analysis over time. Compared to the EMF analysis, the hydraulic pressure acted uniformly on the workpiece for an extended period, resulting in shaping along the target shape. This confirms that the bouncing effect, which is typical in high-speed forming technologies, did not occur and that the surface in contact with the die was uniformly shaped. During EMF, the central dead zone causes the Lorentz force to dominate in the intermediate region slightly off-center from the center of the workpiece, hindering the inflow necessary for sufficient forming. In contrast, in the EMHF, the initial part of the forming process starts at the center of the workpiece, allowing the hydraulic pressure to flow continuously until the limits of shaping are reached.

**Figure 16:**
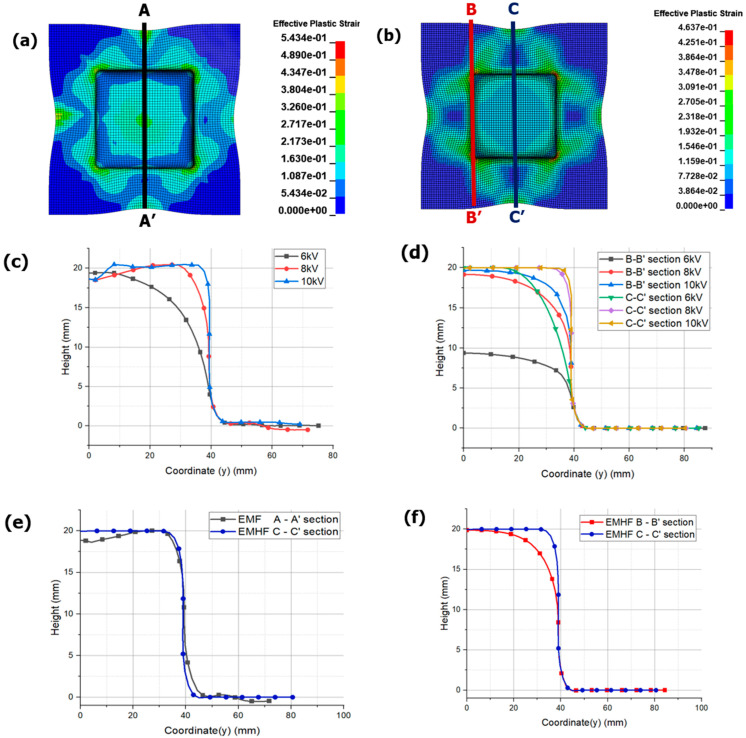
Effective plastic strain of blanks in 8 kV with different sections: (**a**) section marked in EMF, (**b**) section marked in EMHF and different displacement results: (**c**) different voltage condition in EMF, (**d**) different voltage condition in EMHF, (**e**) comparison between EMF and EMHF, and (**f**) comparison between B-B’ and C-C’ in EMHF.

Figure 16 compares the results after the forming convergence of EMF and EMHF. Figure 16a,b depicts top views of Figure 14d and Figure 15d, respectively. The A-A’, B-B’, and C-C’ sections are defined for comparison of each section. Figure 16c compares the results at the A-A’ section for each input voltage. In the case of EMF, incomplete forming was observed at 6 kV. This is because, as shown in Figure 9a, the maximum height obtained under the free-bulge forming condition at 6 kV was 20.065 mm. On the other hand, at 8 kV and 10 kV, although the workpiece enters the die periphery, it is uneven and particularly rough due to the bouncing effect occurring at the center, highlighting the limitations of EMF in achieving smooth surfaces during sheet metal forming. In contrast, the results of EMHF are superior compared to those of EMF. However, in the case of B-B’, forming did not occur at the corners of the die. This suggests that forming was incomplete because of the decrease in hydraulic pressure before a strong forming force could be continuously transmitted to the corners. To shape the corners, a higher applied pressure and improved die shape are required. Additionally, the fact that the forming angle in the current model was perpendicular may have contributed to this issue. Nevertheless, the forming quality was considered excellent compared with the results obtained from the EMF analysis.

### 3.3. Asymmetry Die Analysis

**Figure 17:**
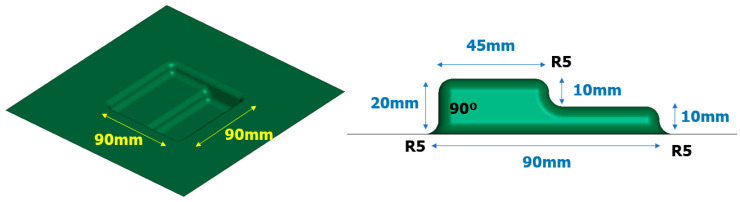
Shape of the asymmetry die for EMHF analysis.

A blank was performed on the forming potential of EMHF not only for symmetric die shapes but also for asymmetric die shapes (Figure 17). The EHF technique generates a shaping force from the plasma, causing instantaneous explosions at the center of the chamber. However, during forming, empty spaces may occur inside the chamber and the explosive energy dissipates, leading to a lack of continuous hydraulic action, posing significant challenges in the use of asymmetric dies. We attempted to address this by applying the EMHF technique.

**Figure 18:**
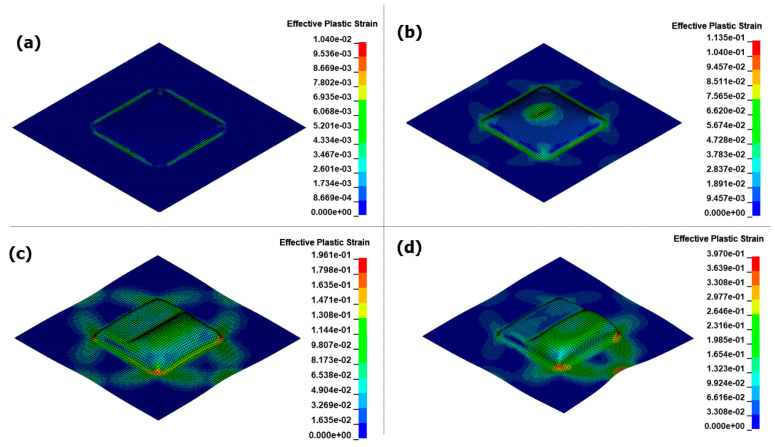
Effective plastic strain for EMHF in different times with 8 kV input voltage: (**a**) 0.4 ms, (**b**) 0.615 ms, (**c**) 0.78 ms, and (**d**) 1.35 ms.

Figure 18 shows the results of the EMHF analysis over time. In the initial 10 mm of forming, the fluid distributes the load symmetrically. However, as time progresses, forming requires an asymmetric shape, and the inlet of the workpiece varies. Notably, no bouncing effect was observed, and some forming was possible even with irregular shapes. However, the inlet required by the die was asymmetric, with more inlets occurring when higher forming heights were required.

**Figure 19:**
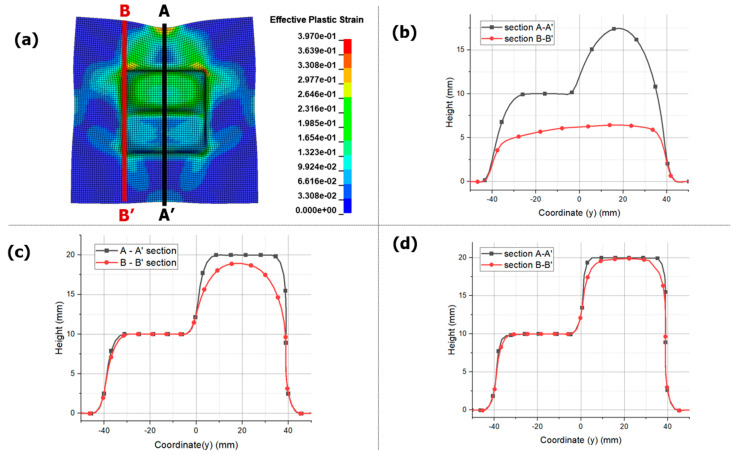
Height of blanks in EMHF with different sections: (**a**) effective plastic strain values in 8 kV (**b**) 6 kV, (**c**) 8 kV, and (**d**) 10 kV.

Figure 19 shows a graph comparing the degrees of forming in the central and outermost regions of the workpiece. The section marked A-A’ represents the central region of the workpiece, which is symmetric about the center and achieves the maximum possible forming height. Each die had heights of 10 and 20 mm, resulting in uniform forming and no bouncing effect on the surface. However, section B-B’ showed significant differences, particularly in areas with varying gaps. While forming closely followed the target shape at a height of 10 mm, it did not reach the desired shape at the maximum height of 20 mm. This suggests a hydraulic energy loss and localized load concentration, similar to a rectangular die. Therefore, improvements are necessary, particularly for forming sharp edges in corner sections.

## 4. Conclusions

In this study, we compared and analyzed a conventional EMF process with a hybrid EMHF process. For the free-bulge die, the forming time for EMF was approximately 0.5 ms, indicating a relatively rapid forming process. However, uneven forming occurred because of the influence of the dead zone in the forming coil. Additionally, it was observed that forming did not occur in SUS430. In contrast, the EMHF was not influenced by the electrical conductivity of the workpiece and showed improved formability compared with the EMF method under the same applied voltage. While the maximum strain rate during EMF forming was approximately 4850/s, EMHF forming occurred in a lower range of 1250/s. The forming convergence time was also approximately 1.4 ms. This suggests a momentary reduction in the material’s deformation speed owing to the conversion of the fluid and punch momentum; however, it is still considered a sufficiently high-speed forming method.

In addition to the free-bulge shape, an application was performed for rectangular and asymmetrical shapes. During EMF, the high-speed motion of the workpiece caused by the Lorentz force led to a bouncing effect upon collision with the die. This resulted in uneven or incomplete forming. However, with EMHF, the forming time was generally longer, allowing for a more uniform application of the load to the workpiece over an extended period compared with EMF. However, it is believed that the forming limitations in localized areas are not solely due to the technical constraints of EMHF, but rather a common issue in sheet metal forming. It is considered an aspect that can be improved through further research and development efforts in the future.

In this study, we analytically reviewed the new process of EMHF and confirmed its potential as a sheet metal forming technique. We plan to further investigate its applicability to various forming processes that conventional high-speed forming techniques such as EMF and EHF have not been able to address. From the analysis results of EMHF, it was observed that forming was not well achieved at sharp R sections for a specific die configuration. We intend to assess the forming feasibility of these sections through future research endeavors.

## Figures and Tables

**Table 1:** Parameters of quasi-static material properties for Al5052 and SUS430.

	A (MPa)	B (MPa)	n
Al5052	188.03	327.07	0.58
	**K (MPa)**		n
SUS430	689.74		0.21

**Table 2:** Parameters of dynamic material properties for Al5052 and SUS430.

	c	p
Al5052	4232.7	22.5
SUS430	76.26	5.0

## Data Availability

Data are contained within the article.
